# Cardiac POCUS in Pediatric Emergency Medicine: A Narrative Review

**DOI:** 10.3390/jcm12175666

**Published:** 2023-08-31

**Authors:** Eric Scheier

**Affiliations:** 1 Pediatric Emergency, Kaplan Medical Center, Rehovot 76100, Israel; ericsc@clalit.org.il; Tel.: +972-(8)-944-1275; Fax: +972-(8)-944-1276; 2 Faculty of Medicine, Hebrew University of Jerusalem, Jerusalem 9112001, Israel

**Keywords:** cardiac, pocus, ultrasound, pediatric

## Abstract

Purpose of this Review: The cardiac point of care ultrasound (POCUS) is among the most impactful examinations in the evaluation of an ill child. This paper will review the English-language literature on cardiac POCUS in the pediatric emergency department (PED), the adult emergency literature with relevance to pediatric emergency, and other pediatric cardiac studies outside pediatric emergency with relevance to PED detection of potentially emergent pediatric cardiac pathology. Recent findings: Pediatric emergency physicians can reliably detect decreased left-sided systolic function and pericardial effusion using POCUS. Case reports show that pediatric emergency physicians have detected right-sided outflow tract obstruction, aortic root dilatation, and congenital cardiac disease using POCUS. Training for pediatric cardiac POCUS competency is feasible, and cardiac POCUS does not increase the burden on cardiology resources to the PED. Summary: While cardiac pathology in children is relatively rare, pediatric cardiac POCUS can incorporate a broad curriculum beyond systolic function and the presence of pericardial fluid. Further research should assess pediatric emergency physician performance in the identification of a broader range of cardiac pathology.

## 1. Introduction

The cardiac point of care ultrasound (POCUS) is among the most impactful examinations in the evaluation of an ill child as early identification of cardiac emergency can lead to tailored therapy that can be lifesaving. For example, a child with wheezing and presumed asthma may worsen with liberal fluid and beta-agonist therapy until the underlying myocarditis is discovered. Moreover, congenital heart disease is a leading cause of perinatal and infant mortality [1]. Even in older children, the identification of cardiac disease can tailor therapy. As detailed in Kehrle et al., the identification of a cardiac lesion using POCUS for a fifteen-year-old in cardiac arrest led to the early initiation of extracorporeal membrane oxygenation [2].

Cardiac POCUS is one of the few applications to be used in pediatric anesthesia, critical care, and neonatology as well as in pediatric emergency medicine [3,4,5]. Cardiac POCUS training for pediatric emergency physicians is supported by the American Academy of Pediatrics, the American College of Emergency Physicians, and the American Society of Echocardiography [6,7,8]. In a recent study, over half of cardiac POCUS in the PED was performed to evaluate chest pain with approximately 20% performed to evaluate dyspnea and tachycardia, respectively, and 10 percent to evaluate syncope [9].

Pediatric hearts are on the whole anatomically intact and function at peak performance, while adult hearts are more likely to show atherosclerotic changes [10]. Therefore, pediatric cardiac emergencies tend to develop from respiratory emergencies, inborn errors in electric conduction, and infectious myocarditis or from more indolent processes such as ventricular septal defects that can cause failure over time. Unique to the pediatric patient is the abrupt change in physiology that occurs in early infancy with the closure of the ductus arteriosus. Thus, pediatric cardiac emergencies can involve unfamiliar anatomy and global failure, while adult cardiac emergencies are more likely to involve regional ischemia and diastolic dysfunction from scarring.

The scope of cardiac POCUS application in children is generally limited to the identification of life-threatening pathology. The breadth of the literature discussing this examination in the pediatric emergency department (PED) is relatively limited in comparison with other pediatric POCUS examinations and with the adult cardiac POCUS literature. This is a narrative review of the English-language literature describing the use of cardiac POCUS in pediatric emergency medicine, the adult emergency literature with relevance to pediatric emergency, and other pediatric cardiac studies outside pediatric emergency with relevance to PED detection of potentially emergent pediatric cardiac pathology.

Studies were collected by searching the terms ‘pediatric, cardiac, pocus, ultrasound, bedside, focus, point of care’ in Google Scholar and Pubmed followed by reviewing relevant studies. This review will examine the existing literature on cardiac POCUS in children in the context of pediatric emergency medicine and suggest areas for future research.

## 2. Cardiac POCUS Examination Technique

Traditionally, the examination incorporates parasternal long-axis parasternal short-axis, apical four-chamber subcostal, and subxiphoid views, with a long-axis view of the inferior vena cava as it enters the right atrium. A full description of the technique is not within the scope of this review but can be found elsewhere [11,12]. The emergency medicine literature recommends that the POCUS examination assess the “five Es”: “presence of an effusion, the degree of variation in diameter of the entrance (inferior vena cava) with respiration, left ventricular ejection, the diameter of the exit (aortic root), and equality in size of the ventricular chambers” [13].

While a right-screen probe marker is used most often in echocardiography, most PEM-performed POCUS examinations place the probe marker on the left side of the screen. However, the convention is institution-specific. The parasternal long-axis single-view is highly sensitive and specific for identifying left ventricular systolic function and moderate to large pericardial effusion [14]. In a quality improvement study in which cardiologists reviewed clips obtained by PEM physicians, the apical four view was noted to have the lowest level of quality and interpretability [15].

## 3. Applications

### 3.1. Cardiac Arrest

Su et al. described the extreme modality of needle insertion into the left ventricle performed in 1893. The stillness of the needle was presented as evidence of cardiac standstill and death [16]. Cardiac POCUS is a more updated and accurate modality to assess cardiac activity and has been used in resuscitations for this purpose.

While the demonstration of cardiac standstill has utility in the management of pediatric cardiac arrest [17], it is problematic to use cardiac standstill as an indication to halt resuscitation efforts as the return of spontaneous circulation after cardiac standstill in children has been reported [18]. Ventricular fibrillation without any meaningful cardiac anterograde flow can be interpreted as a standstill, and clot formation in the left ventricle is evidence that the standstill is irreversible. Valve fluttering from fluid boluses and cardiac movement from positive pressure ventilation should not be interpreted as intrinsic cardiac activity [16]. In practice, the literature defines cardiac standstill as absent myocardial motion, but it shows a low level of interobserver agreement in the evaluation of cardiac standstill [19]. In a study of 263 PEM attendings with cardiac POCUS experience, agreement regarding cardiac akinesis was highest when wall motion was accompanied by valve motion (kappa = 0.740) and lowest when wall motion was not accompanied by valve motion (kappa = 0.304) [20].

In an adult cardiac arrest simulation study, the parasternal long view was found to have a shorter time to acquisition and higher image quality when compared with the subxiphoid view [21].

Leviter et al. presented two cases in which pulse checks during resuscitation were demonstrated sonographically in children where the chest was obscured with defibrillator pads or automated compression. Organized cardiac activity was demonstrated from either the apical-four window or over femoral vessels [22]. In a study of 22 pediatric volunteers, Leviter et al. showed that occlusion of the parasternal window with defibrillator pads could be overcome using apical four-chamber and subxiphoid views and that femoral pulse check could be performed with POCUS in the same time frame as a conventional pulse check with good agreement between novice and expert sonographers. There, 86% of apical four and 94% of subxiphoid clips were interpretable for contractility on the first attempt, and 94% of apical four and 100% of subxiphoid on the second attempt. Femoral artery pulsation was visible in 70% of views on the first attempt and 80% of views on the second attempt [23].

### 3.2. Systolic Ventricular Failure

Following case reports of systolic failure identified using POCUS [24,25], Pershad et al. showed a 4.4% difference between PEM physicians and pediatric echocardiographers in the measurement of shortening fraction in children admitted to the intensive care unit [26].

While assessment of cardiac function is frequently performed qualitatively, advanced practitioners can use quantitative measures such as end-point septal separation (EPSS, a measure of the distance at end-diastole between the distal point of the anterior leaflet of the mitral valve and the ventricular septum), fractional shortening, and the mitral annular plane systolic excursion (MAPSE), although EPSS has only been validated in adults [5]. Normal EPSS is below 5 mm; EPSS above 7 mm indicates reduced ejection fraction in adults [27]. Schick et al. found a ROC for MAPSE for left systolic failure of 0.79 with interrater reliability of 96% and concluded that a MAPSE value less than 8 mm had moderate predictive value and specificity for a left ventricular ejection fraction of <50% [28]. Interestingly, a study by Bahle et al. showed a poor correlation between fractional shortening and EPSS when compared with formal echocardiography. There, they found a much higher correlation between visual estimation of systolic dysfunction by experienced physicians and echocardiography (kappa 0.94–0.97) [29]. Bustam et al. showed that emergency medicine trainees could also visually identify systolic failure accurately after 3 h of practical training [30].

Similar to MAPSE, emergency physicians can use the tricuspid annular plane systolic excursion (TAPSE) to assess changes in right ventricular systolic function [31].

Longjohn’s study of 171 pediatric patients presenting with arrest, shock, or cardiomegaly on radiograph is over ten years old and showed a kappa of 0.87 between pediatric emergency physicians and echocardiographers blinded to the clinical scenario. Included in this series was a child with an ECG that showed only sinus tachycardia, underscoring the importance of POCUS in the early evaluation of cardiac activity [32]. Puri et al. reported 191 cases of pediatric heart failure in children with no known cardiac disease; 49% were missed at first presentation, most often with gastrointestinal symptoms [33]. Scott et al. reported a median time to POCUS of 33 min in the diagnosis of pediatric heart failure [34]. Miller et al. reported a sensitivity and specificity of 100% and 99.5%, respectively, in 456 real-time PED cardiac POCUS examinations for both pericardial effusion and left ventricular systolic dysfunction when compared with expert POCUS review [9]. However, Miller’s study primarily used qualitative evaluation (mild, moderate, severe) rather than quantitative assessment of function.

Hamad et al. presented a series of ten pediatric cases demonstrating biventricular heart failure [35]. In Hamad’s series, the BNP was normal in two patients with fulminant myocarditis and troponin was normal in one, again underscoring the value of early POCUS. Troponin can be elevated in non-failure processes such as sepsis, and early POCUS may be useful to confirm adequate contractility.

Although without the b-mode component of cardiac POCUS, Stewart et al. reported the accuracy of the ultrasound cardiac output monitor, a Doppler-based ultrasound modality recorded at the suprasternal notch in the assessment of pediatric stroke volume and cardiac output [36].

Bracy et al. found that emergency physicians could identify regional wall motion abnormality in patients with acute coronary symptoms [37]. There, cardiac POCUS was performed with parasternal long, parasternal short, apical four-chamber, and apical two-chamber views to image 13 regional wall segments. While acute cardiac syndrome is an adult presentation, wall motion abnormality in children may be the first sign of impending systolic failure in myocarditis.

### 3.3. Diastolic Failure

Diastolic heart failure (DHF) is defined as heart failure with normal (or near-normal) left ventricular ejection fraction, in the absence of other explanatory conditions such as valvular lesions, and can be identified in adults using ED POCUS. Diastolic dysfunction is assessed using both tissue Doppler evaluation of the mitral valve annulus and pulsed wave Doppler measurement of flow across the mitral valve into the left ventricle. Tissue Doppler lengthening velocities are denoted by e` and a`, respectively, while mitral valve flow velocities are denoted by E and A, respectively. Diastolic function is considered to be abnormal in adults if the septal e` is less than 8 cm/s and if the lateral e` is less than 10 cm/s. In cases of discordance of septal and lateral interpretations, an E/e’ ratio of less than 8 is considered normal [38]. Both Ünlüer et al. and Ehrman et al. defined severe diastolic dysfunction as an early to late peak trans-mitral inflow velocity diastole velocity ratio above 1.5 [38,39]. Agreement among emergency medicine physicians in the assessment of diastolic dysfunction in the apical four view was good, with the caveat that too few patients were recruited to properly power the kappa study [40]. Lin et al. found moderate agreement (kappa = 0.67 when examinations were spaced no more than 3 h apart) between emergency physicians and study cardiologists in the assessment of diastolic dysfunction [41]. Ehrman added septal and lateral mitral annular excursion velocity in early diastole using tissue Doppler imaging as per echocardiography recommendations but found that emergency physicians were less proficient in this modality [39]. There is no literature on pediatric diastolic dysfunction identified on POCUS.

### 3.4. Pericardial Effusion and Tamponade

Pericardial fluid is seen first as an anechoic stripe deep to the parietal pericardium at the most dependent portion of the pericardial sac and becomes circumferential as fluid accumulates [42]. Acute pericarditis diagnosis should have at least two of the four criteria according to the 2015 ESC guidelines of pericardial diseases, which are characteristic chest pain (sharp pain), pericardial friction rub, pericardial effusion in echocardiography, and typical electrocardiogram changes (concave ST-segment elevation and PR-segment depression or elevation) [43]. Longjohn, as per above, showed that POCUS can accurately diagnose pericardial effusion in children [32]. The majority of studies using cardiac POCUS to identify pericardial effusions and tamponade in the pediatric ED setting are limited to case reports [44,45,46]. Tsang et al. showed that pediatric patients who had ultrasound-guided pericardiocentesis had a 99% success rate, with 93% on the first attempt, and 1% major and 3% minor complication rates [47].

Tamponade is defined as the presence of an effusion causing right ventricular diastolic collapse, right atrial systolic collapse, a plethoric IVC with lack of respiratory variation, and an exaggerated decrease in inspiratory mitral valve inflow velocity on pulsed wave Doppler [44]. Ten percent of patients with pericardial tamponade will have neither jugular venous distension, muffled heart tones, or hypotension [45]. The first case report on tamponade in a child is over twenty years old [47], with additional cases more recently [45,48]. Bergmann et al. reported six pediatric cases of tamponade that presented without hypotension [42]. They demonstrated tamponade by placing the m-mode line over both ventricles in the long axis and demonstrated right ventricular collapse early in diastole, just after the E wave. Right atrial collapse during systole similarly indicates tamponade with higher sensitivity but less specificity [44]. Alerhand et al. noted that tamponade physiology can also be demonstrated with sonographic pulsus paradoxus, the variation in flow velocity through the mitral valve with respiration when viewed in apical four on m-mode [49]. The size of a pericardial effusion can be measured with the myocardium–epicardium diameter in diastole. A small effusion corresponds to a diameter of <10 mm, moderate corresponds to 10 to 20 mm, and large corresponds to >20 mm. Pericardial effusion, seen anterior to the descending aorta on long-axis view, should not be confused with pleural effusion, which lies posterior to the descending aorta. Further, pericardial effusion is diffusely anechoic in contrast with a pericardial fat pad, which appears stippled [11].

Evaluation of the inferior vena cava (IVC) is not covered in this review, as the majority of the pediatric literature on POCUS evaluation of the IVC is in the context of fluid balance. A plethoric IVC, defined as an IVC diameter larger than 2.1 cm with less than 50% inspiratory variation is up to 97% sensitive to tamponade [11].

### 3.5. Right Ventricular Outflow: Pulmonary Embolus

No definitive studies or clinical prediction scores have established a role for D-dimer testing in children. Rather, ventilation/perfusion (V/Q) scanning and helical CT are indicated to assess for PE. Presley et al. reported a case of pulmonary embolus detected by identifying an enlarged right ventricle (RV:LV ration > 1) and plethoric IVC. While these findings are not pathognomonic, they may be used to guide further evaluation [50].

### 3.6. Right Ventricular Outflow: Pulmonary Hypertension

Several case reports demonstrate that POCUS can expedite the diagnosis of pulmonary hypertension [51,52,53]. Pulmonary hypertension can be demonstrated by dilation of the right ventricle and/or the right atrium, flattening of the interventricular septum, a hypertrophic right ventricular free wall, and decreased right ventricular function, when severe [53]. An additional method to identify pulmonary hypertension is tricuspid jet flow velocity, which measures flow from the right ventricle retrograde into the atrium in the apical-four view. A velocity above 2.5 m/s is indicative of pulmonary hypertension.

### 3.7. Left Ventricular Outflow, Aortic Dissection

Ninety percent of patients with aortic dissection present with dilation of the aorta greater than 4 cm at the time of diagnosis [54]. There is a significantly decreased time to diagnosis of over two hours with the use of POCUS in the evaluation of the ascending aorta. Of 13 cases with a CT angiography aortic root measurement of over 4 cm, 10 were identified using ED POCUS and none were missed by the emergency physician who performed POCUS [54]. While the pediatric literature does not report on POCUS identification of aortic dissection, a case report exists on aortic dissection identified using POCUS on a 21-year-old male. There, authors note that aortic dissection in young adults is often associated with collagen disorders such as Marfan syndrome, congenital bicuspid aortic valve, vasculidities, and cocaine abuse [55]. Therefore, PEM physicians should be aware of this potential diagnosis in teenagers. Of note, a visualized dissection flap and signs of pericardial effusion with tamponade on sonography were shown to have a higher incidence of rupture, and patients with these signs should go directly to the operating room without waiting for additional imaging to be performed.

### 3.8. Left Ventricular Outflow: Hypertrophic Obstructive Cardiomyopathy (HOCM)

HOCM is defined as septal-to-posterior wall thickness in end-diastole above 1.5:1 in hypertensive patients or above 1.3:1 in non-hypertensive patients, with a maximal wall thickness in any segment of more than 15 mm. Metabolic and infiltrative cardiac disease, in contrast, may present with concentric hypertrophy [11]. Routine cardiovascular screening examinations are neither sensitive nor specific for HOCM [56]. Zhang et al. presented a case series of HOCM on POCUS in adults with a minimal myocardial diameter of 21 mm in the series [57]. One of the cases presented involved ventricular hypertrophy as a result of aortic stenosis. In that case, they note that the blood flow acceleration point as measured using color Doppler should be located in the aortic valve level rather than more proximally in the left ventricle.

While performed outside the emergency department, Fox et al. showed that medical students can screen for HOCM in high school athletes. In total, 2332 students were screened, of whom 5.8% were flagged by students as abnormal and 5.1% confirmed on echocardiography to be abnormal [58].

## 4. The Effect of POCUS on the Frequency of Cardiology Referrals

In a pediatric population, Singh et al. reported no significant difference in echocardiography (42 vs. 46), cardiology consultations (36 vs. 37), or cardiology referrals (47 vs. 37) after the introduction of cardiac POCUS. However, the number of children with cardiology pathology found using POCUS (five instances) and formal echocardiography (one major finding: mildly reduced left ventricular systolic function) was low [59]. There, 58 of 65 (89%) POCUS examinations were felt to be interpretable for assessment of global biventricular systolic function and effusion by the secondary reviewer. Of the 65 scans reviewed, a single major discrepancy was identified. This scan was initially interpreted as normal global biventricular systolic function and no pericardial effusion but was found to demonstrate severely reduced left ventricular systolic function on secondary review.

## 5. The Route to Competency in Cardiac POCUS

A number of studies demonstrate that competency in cardiac POCUS is achievable without extensive training. Chisohm et al. reported increased competency in adult cardiac POCUS after 10 h of mixed didactic training and more than 45 ultrasounds [60]. Pershad’s pilot study required investigators “cumulative of 2 h of reading material and didactic instruction, 5 proctored examinations with a pediatric echocardiography technician, and 10 examinations performed on healthy volunteers and critiqued by the pediatric cardiologists” [25]. Miller required “a single didactic one-hour lecture, performance of 25 educational cardiac POCUS examinations that are reviewed by POCUS experts for adequacy of study clips and accuracy of interpretation, followed by a proctored examination” [9] and state that the 25 studies were based on Gaspar’s recommendation of 24 proctored studies to achieve competency [61].

Pediatric cardiac POCUS education has been incorporated into pediatric residency education. Hulse et al. trained 49 first-year pediatric residents in various POCUS applications. The cardiac module was composed of a lecture followed by three hours of precepted training. Comfort acquiring cardiac images increased from 18% to 86% before and after the module [62]. Kwan et al. used a web-based series of cases and recorded the number of cases required to achieve an accuracy of 90%. For cardiac images, 239 of 279 participants reached the benchmark accuracy at an average of 128 cases [63]. Normal function was the easiest to recognize, followed by pericardial fluid, poor cardiac function, and dilated or hypertrophied ventricle.

Riera et al. performed a quality improvement study in conjunction with their cardiologists in order to improve the quality and quantity of cardiac POCUS studies performed in the PED. Over the course of their study, they increased the number of studies six-fold. Within three months of initiating the project, they had a four-month interval in which all parasternal long-axis clips were of adequate quality [15].

While not performed in the context of pediatric emergency medicine, several studies examined parent performance with cardiac POCUS and may be relevant in the context of home identification of cardiac emergencies. Jaji et al. used a 25-min workshop to teach lay-parents to acquire parasternal-short and apical four views of the heart [64]. Fractional shortening and ejection fraction were calculated using these images and compared to recent measurements acquired on echocardiography. Included were children (median age, nine months) followed by the pediatric cardiology service who had normal ventricular anatomy. In total, 24 of 25 parasternal short-axis images and 24 of 25 apical-four images were adequate for the assessment of cardiac function. There was a moderate but significant (r = 0.62) correlation between fractional shortening acquired by parents and fractional shortening acquired on echocardiography.

Chen et al. performed a similar study looking at parental assessment of the aortic root in Marfan syndrome. Fifteen parents reviewed a training video prior to home measurement of their child’s aortic root. Aortic root measurement differed from echocardiography by a range of 0.02 to 0.24 cm [65]. Voleti et al. taught six laypeople a simplified screening protocol in which a mitral regurgitation jet of ≥1.5 cm and/or the presence of aortic insufficiency were considered a positive screen for rheumatic heart disease in a pediatric population in the Republic of Palau. In total, 4.1% of children screened were diagnosed with borderline or definite rheumatic heart disease with a mean sensitivity and specificity of 71% compared with expert care [66]. Ploutz et al. performed a similar study using nurses and handheld sonography in Uganda [67]. There, the nurses had prior experience with limited echocardiography. Each nurse received four hours of physician-directed teaching followed by two days of training. Each nurse performed and interpreted a minimum of 50 supervised studies over this period. The nurses had a sensitivity of 74.4% and a specificity of 78.8% in the detection of any rheumatic heart disease.

## 6. Limitations

This review is limited by the fact that it is a narrative rather than a systematic review and was composed by a single author. The reviewed studies were heterogeneous, and the majority were performed in a general emergency department rather than in a pediatric population. The level of evidence in pediatrics is therefore low and consists largely of case reports.

## 7. Conclusions

Pediatric emergency physicians can identify systolic failure and pericardial effusion. The pediatric literature lacks discussion of diastolic dysfunction, regional wall motion abnormality, and aortic dissection. Pulmonary embolus, pericardial tamponade, pulmonary hypertension, and various cardiac anomalies are described in children as case reports only. Training for pediatric cardiac POCUS competency is feasible, and cardiac POCUS does not appear to increase the burden on cardiology resources to the PED.

Further research should measure time to competence in more advanced pediatric cardiac applications such as the identification of wall motion asymmetry as has been performed in the adult emergency literature. Further research may also investigate screening examinations for HOCM and ventricular septal defects as an extension of PED physical examination, and home identification of cardiac emergency by laypeople. For example, a child with a working diagnosis of bronchiolitis per phone triage assessment may be able to be diagnosed with myocarditis at home, thereby facilitating referral to an appropriate facility.

## Data Availability

Not applicable.
